# Novel multilayer TiO_2_ heterojunction decorated by low g-C_3_N_4_ content and its enhanced photocatalytic activity under UV, visible and solar light irradiation

**DOI:** 10.1038/s41598-019-42438-w

**Published:** 2019-04-11

**Authors:** Yizheng Wang, Jiang Yu, Weidong Peng, Jing Tian, Chun Yang

**Affiliations:** 1 College of Architecture and Environment, Sichuan University, Chengdu, 610065 China; 2 Institute of New Energy and Low Carbon Technology, Sichuan University, Chengdu, 610065 China; 3 College of Chemistry and Materials Science, Sichuan Normal University, Chengdu, 610068 China; 4 Computational Visualization and Virtual Reality Key Laboratory of Sichuan Province, Chengdu, 610068 China

**Keywords:** Photocatalysis, Nanoscale materials

## Abstract

In this paper, we used a facile ball milling, microwave radiation and heating treatment method to achieve the surface modification of TiO_2_ with low g-C_3_N_4_ concentration, and a multilayer heterojunction composite with TiO_2_ as the main part and g-C_3_N_4_ as the modification agent was obtained. The obtained materials were analyzed by several characterizations to get information on their chemical composition, crystalline structure, vibrational features and optical properties. The photocatalytic performance was evaluated by degradation of rhodamine B (RhB) and methylene blue (MB) under UV, visible and direct solar light irradiation. Its photocatalytic activity was enhanced depended on the novel structure of g-C_3_N_4_/TiO_2_ hybrid and the special Z-scheme electron-hole transfer model of multilayer heterointerfaces. The material preparation and structural features could be useful for the design and development of other photocatalysts with high photocatalytic activity.

## Introduction

Photocatalysis has been attracting great attention due to increasing energy demands and serious environmental pollution in recent years^1–7^. As an important non-metallic semiconductor photocatalyst, graphitic carbon nitride (g-C_3_N_4_) has been widely used in photocatalytic sterilization, hydrogen production, pollutant degradation, and many other fields^8–11^, due to its high stability, unique electronic structure, and narrow band gap (about 2.7 eV) that widens its light response to visible region of 450 nm^12^. However, g-C_3_N_4_’s low yield and high e^−^h^+^ pair recombination rate limit its practical application^13^. On the other hand, titanium dioxide (TiO_2_) is widely used in industry as a photocatalyst due to its high specific surface area, large pore volume, strong oxidizing power, low cost, and high chemical stability^14,15^. But its’ narrow light-response range and low quantum efficiency may restrict the practical applications^15^. A new composite that combines g-C_3_N_4_ and TiO_2_ might integrate their advantages, favor the separation of e^−^h^+^ pairs, and improve the photocatalytic performance, which could become a new material with industrial application prospect.

Currently, most of the g-C_3_N_4_/TiO_2_ composites are mainly prepared by hydrothermal method^16–18^, calcination process^19–21^, solvent evaporation method^22^, solvothermal strategy^23^, etc. Gu *et al*.^16^ developed the flower-like TiO_2_/g-C_3_N_4_ hybrid by surfactant-assisted hydrothermal treatment. Hao *et al*.^19^ fabricated the g-C_3_N_4_/TiO_2_ heterojunction photocatalysts that preserved a macroscopic network structure with a relatively regular array of macropores by a calcination method using tetrabutyl titanate and melamine as the feedstocks. The morphology of g-C_3_N_4_/TiO_2_ hybrids is also varied via different preparing methods, such as flower-like^16^, hollow nanobox^23^, hollow core@shell structure^24^, corn-shaped structure^17^, multilayered structure^25^, etc.

Table 1 lists several typical g-C_3_N_4_/TiO_2_ hybrid photocatalysts that were prepared using different methods. The g-C_3_N_4_/TiO_2_ hybrids with relatively high contents of g-C_3_N_4_ have been studied in recent years, however, little has been known about g-C_3_N_4_/TiO_2_ hybrids with low contents (less than 10 wt%) of g-C_3_N_4_. What is the structure and photocatalytic performance of g-C_3_N_4_/TiO_2_ hybrids with low content of g-C_3_N_4_? Could this type of photocatalysts form a heterostructure and enhance photocatalytic efficiency? Further exploration of the structure and photocatalytic properties of the g-C_3_N_4_/TiO_2_ hybrid with low g-C_3_N_4_ content (less than 10 wt%) is necessary.

**Table 1:** Optimal g-C_3_N_4_ mass ratio of several typical g-C_3_N_4_/TiO_2_ hybrids.

Hybrids	Preparation method	Target pollutants/products	Light source	Optimal g-C_3_N_4_ mass ratio (wt%)	Ref.
g-C_3_N_4_/TiO_2_	Single-pot microemulsion method	MO,MB,RhB	300 W Xe lamp > 420 nm	17.1	^16^
g-C_3_N_4_/TiO_2_	Annealing method	RhB	350 W Xe lamp > 420 nm	28.3	^19^
g-C_3_N_4_/TiO_2_	Annealing method	AO7	500 W tungsten halogen lamp > 420 nm	12	^20^
g-C_3_N_4_/TiO_2_	Annealing method	NO	500 W Xe lamp > 420 nm	15	^21^
g-C_3_N_4_/TiO_2_	Annealing method	MB	500 W Xe lamp > 420 nm	33	^25^
g-C_3_N_4_/TiO_2_	Solvothermal method	RhB	500 W Xe lamp > 420 nm	25.9	^48^
g-C_3_N_4_/TiO_2_	Hydrothermal method	Propylene	300 W Xe lamp > 420 nm	30	^49^
g-C_3_N_4_ NS/TNTAs	Electrochemical method	RhB	300 W Xe lamp > 420 nm	60	^40^
g-C_3_N_4_/TiO_2_	Hydrothermal method	Phenol	500 W Xe lamp > 420 nm	66.7	^54^
br-TiO_2_/g-C_3_N_4_	Low-basicity solution chemistry method	MO	300 W Xe lamp > 420 nm	35	^41^
g-C_3_N_4_/TiO_2_	Solegel method	Hydrogen	500 W Xe lamp > 420 nm	67	^61^
g-C_3_N_4_/TiO_2_	Ball milling method	Hydrogen	450 W Xe lamp > 420 nm	50	^62^

Although the g-C_3_N_4_/TiO_2_ hybrids listed in Table 1 were reported better photocatalytic performance, technical difficulties in preparing g-C_3_N_4_/TiO_2_ hybrids still limit its utilization, including high temperature processing, complex chemical reactions, precursor hydrolysis and agglomerate, and difficult control in yield. Surface modification of photocatalytic materials is an important technical mean to improve the absorbability and photocatalytic properties of materials^26,27^. As the ball milling method and microwave-assisted heating method are both simple, effective, low-cost and green technique for industrial production to fabricate composite photocatalysts^28,29^, we combined these two methods to prepare a g-C_3_N_4_/TiO_2_ composite with low content (less than 10 wt%) g-C_3_N_4_. In addition, the structural properties and photocatalytic performance of the new g-C_3_N_4_/TiO_2_ composite was studied in this work, which have not been reported by others.

## Materials and Methods

### Materials

Melamine powder (Pur. >99.0%) used in the experiments was supplied by Aladdin Chemistry Co. Ltd. Nano TiO_2_ powder (anatase, 100 nm, Pur. >99.8%) was supplied by XuanCheng JingRui photocatalytic material Co. Ltd. RhB, MB, and other chemicals used in the study were purchased from Aladdin Chemistry Co. Ltd. and other China chemical reagent Ltd. All of the chemical reagents were of analytical purity and used without further purification. Deionized water was used throughout this study.

### Preparation of photocatalysts

**Figure 1:**
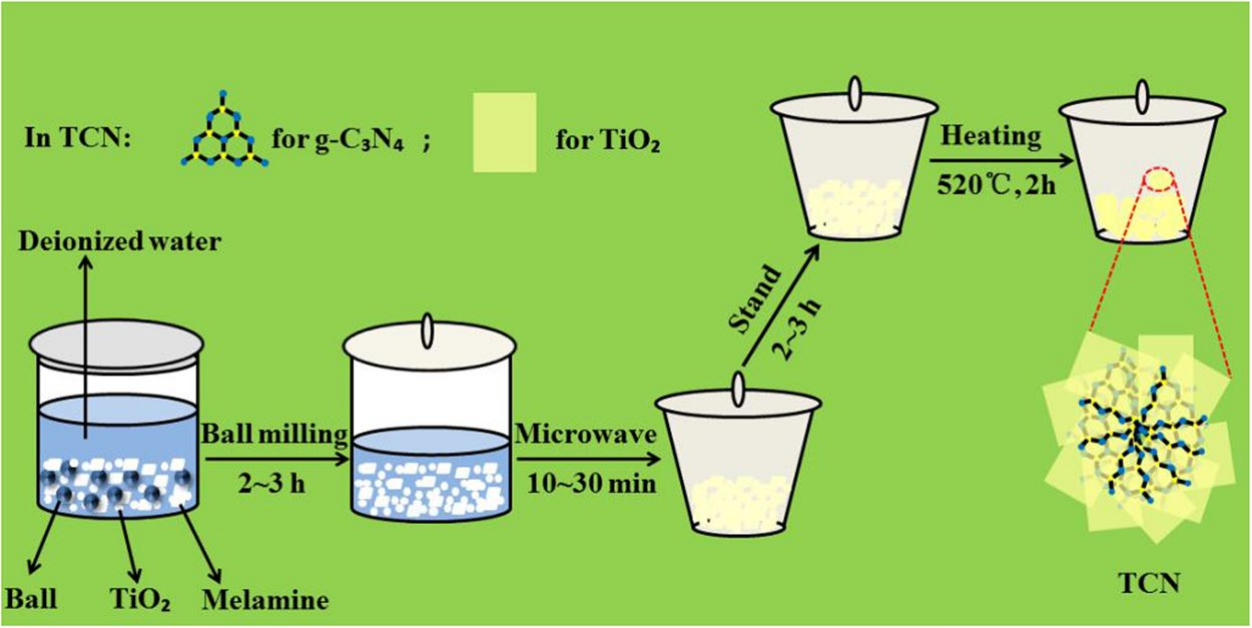
Schematic illustration for the formation of samples.

A combination method of a facile ball-milling, microwave radiation and heating treatment was used in this work. The preparation procedure had four steps (Fig. 1): (1) TiO_2_ powder and a certain amount of melamine were added into a agate ball milling tank (PM2L, Nanjing, China) with a ball: powder ratio of 10: 1, and water was used as a dispersant. The mixtures were milled in the ball milling tank for 2~3 h at a speed of 400 rpm to promote the surface integration of g-C_3_N_4_ and TiO_2_ and to generate a homogeneous heterogeneous interface. (2) The well-mixed wet powder from the ball milling tank was then treated in a microwave oven at 700 W for 10~30 min to get uniform particle size and good compaction. (3) The collected powder was kept for 2~3 h to achieve molecular thermal equilibrium. (4) after that, the powder was heated at 520 °C for 2 h with a heating rate of 5 °C/min in an oven (SX2-2.5-10, Zhengzhou, China). After cooled to room temperature naturally, the final product was collected for further use. By varying the dosage of melamine (30, 40, 50 wt.%), a series of g-C_3_N_4_/TiO_2_ hybrids were obtained, labeled as TCN-1, TCN-2, TCN-3, respectively.

For comparison, pure TiO_2_ was also treated by the above four steps, labeled as TiO_2_-W.

g-C_3_N_4_ was synthesized by directly heating melamine. Typically, a certain amount of melamine powder was poured into an alumina crucible and heated in a muffle furnace at 520 °C for 2 h with a heating rate of 5 °C/min. After cooling to room temperature, the product was collected and ground into powder.

### Characterization

Thermogravimetric analysis (TGA) was performed on a Mettler Toledo TGA/DSC2 (Mettler-Toledo, Switzerland) at a heating rate of 5 °C/min from room temperature to 800 °C under air atmosphere. In order to determine the crystal phase composition and the crystallite size of the photocatalysts, the X-ray diffraction (XRD) patterns of the prepared samples were obtained using an X-ray diffractometer (Empyrean, Shimadzu, Japan) with Cu-K radiation (λ = 0.15405 nm, 60 kV, 60 mA). The samples were scanned in the range 2θ = 10°~80°. The morphology and EDS of the prepared samples were analyzed using a JSM-7500F (JEOL, Japan) scanning electron microscope (SEM). Transmission electron microscopy (TEM) and high-resolution transmission electron microscopy (HRTEM) images were obtained on a FEI Tecnai G20-Stwin electron microscope (FEI, America) to further investigate the morphology and the structure of the catalysts. X-ray photoelectron spectroscopy (XPS) analysis was performed on an Escalab 250Xi system (Thermo Fisher, America). The thickness of the nanosheets and its multilayer heterointerfaces were determined by atomic force microscopy (AFM) (Bruker dimension icon, USA). UV−Vis diffuse reflectance spectra (DRS) measurements were carried out using a UV-3600 (Shimadzu, Japan) spectrophotometer equipped with an integrating sphere attachment. The analysis range was from 200 to 800 nm, and BaSO_4_ was used as a reflectance standard. Photoluminescence (PL) emission spectra were obtained on a Fluorescence Spectrometer (F-7000, Hitachi, Japan). The excitation wavelength was 230–900 nm with the scanning speed of 2400 nm/min. The widths of excitation slit and emission slit were both 5 nm.

### Photocatalytic tests

The photocatalytic activities of as-obtained samples were measured by the degradation of RhB and MB under both UV and direct solar light irradiation. 50 mg of photocatalyst was suspended in 50 mL of an aqueous solution containing 20 mg/L organic dye. Before irradiation, the suspensions were stirred for 60 min in the dark to establish adsorption–desorption equilibrium. To study dye degradation under UV light, a high-pressure 300 W mercury lamp with the radiation of 365 nm was used as UV light source. And the average irradiance measured at the sample was about 500~800 W∙m^−2^ (measured by solar power meter, TES-1333R) and the reactor was directly exposed to sunlight for solar light degradation. A 300 W xenon lamp with a cutoff filter (λ > 420 nm) was used as visible light source to study the degradation efficiency under visible light. At given time intervals, 4 mL of the suspension was sampled to be centrifuged at 10000 rpm for 10 min to remove the particles. Then, the concentration of organic dyes in solution was analyzed using an Alpha-1506 UV–vis spectroscopy (Shanghai Lab-Spectrum Instruments Co. Ltd., China) at the wavelength of 554 nm and 664 nm for RhB and MB, respectively. The degradation for each sample was measured 3 times.

## Results and Discussion

### Characterization of prepared photocatalysts

#### Component analysis

**Figure 2:**
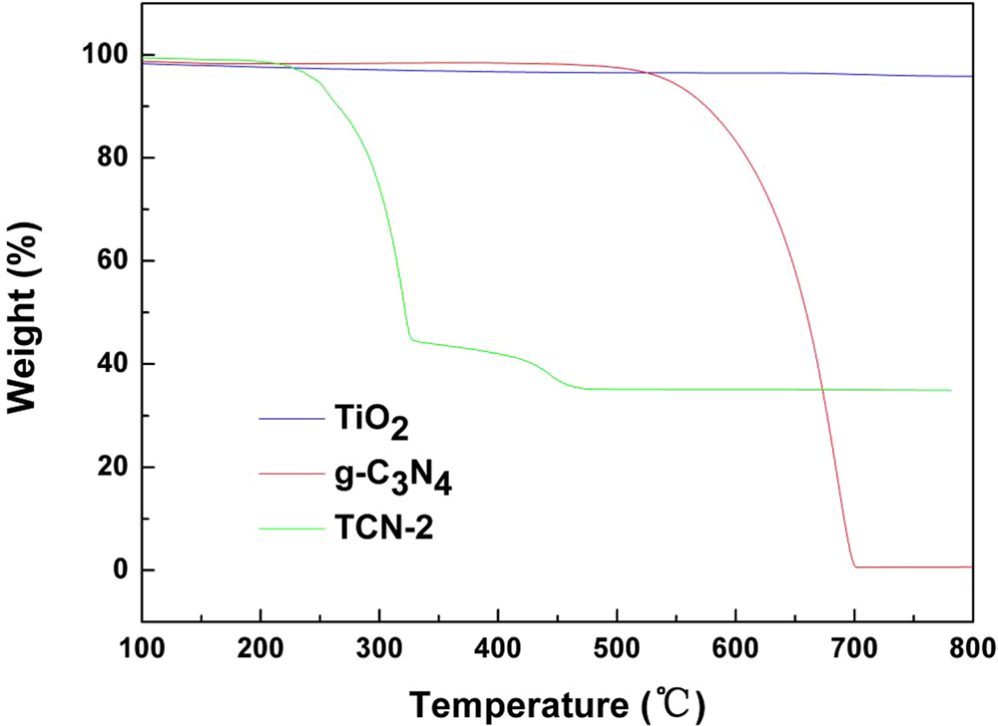
Thermogravimetric analysis curves of anatase TiO_2_, g-C_3_N_4_ and TCN-2 catalyst.

Figure 2 shows TGA analysis results of TiO_2_, g-C_3_N_4_ and TCN-2 photocatalyst. The TGA analysis curve of TCN-2 indicates the existence of g-C_3_N_4_ in the composite. And other reports also confirm that melamine can produce g-C_3_N_4_ at about 500 °C^30–32^. The TCN-2 sample has two weight loss regions: the former weight loss occurred at around 100~300 °C due to desorption of surface bound water and the dehydrogenation and deammonia of melamine to produce g-C_3_N_4_, the later weight loss from 300 °C to 500 °C was due to the combustion of g-C_3_N_4_ in the air atmosphere. And for the TCN-2 sample, the later weight loss is about 8.64% in the temperature range of 300~500 °C, indicating that the TCN-2 sample contains about 8.64% g-C_3_N_4_^19,33^.

**Figure 3:**
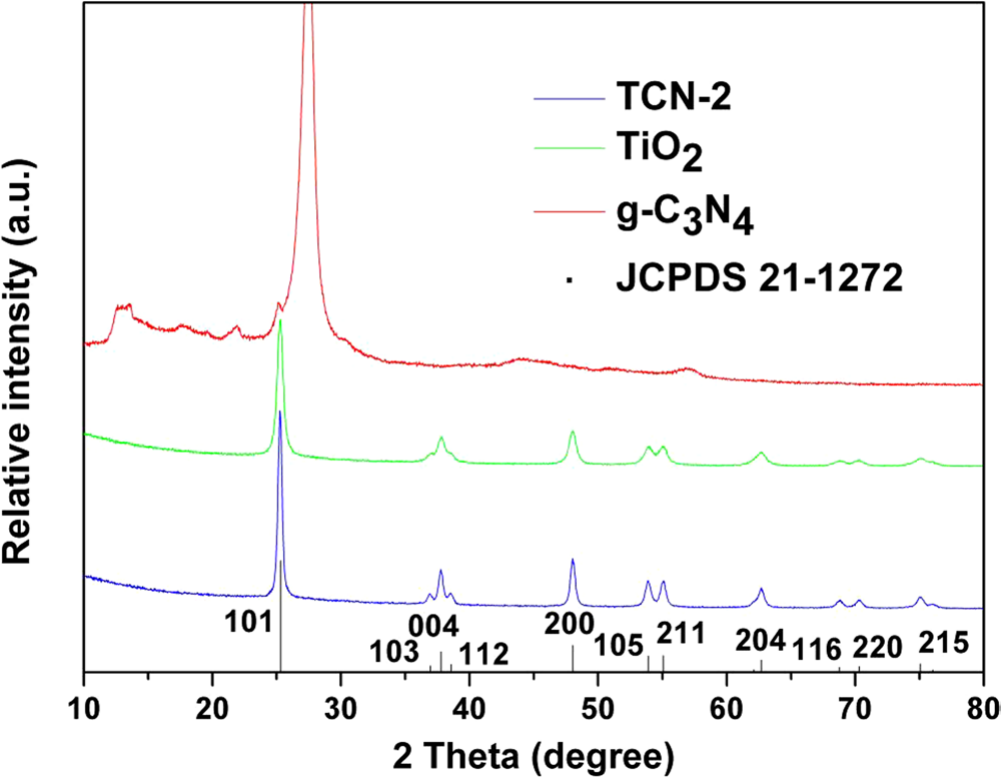
XRD patterns of as-obtained g-C_3_N_4_, TiO_2_ and TCN-2 hybrid.

The XRD patterns of the samples are shown in Fig. 3. For comparison, the XRD patterns for pure TiO_2_ and g-C_3_N_4_ are also given. For g-C_3_N_4_, the main diffraction peak at 2θ = 27.5° indicates its graphite-like structure^34^. Another relative weak peak located at 13.1° may be due to the in-planar repeat period, and just like the hole-to-hole distance among the N-bridged tri-striazine units^35^. It can be seen that the TiO_2_ powder is in good agreement with anatase phase of TiO_2_ indexed to the data in the JCPDS cards (21–1272). No obvious XRD peaks of g-C_3_N_4_ are observed in the TCN-2 hybrid may be due to the small amount of g-C_3_N_4_ whose peaks are not strong enough. These tests show that TiO_2_ is the main part of TCN-2. Furthermore, no obvious XRD peaks of other new crystal phases are found in TCN-2, indicating that the hybridization of TiO_2_ with g-C_3_N_4_ did not change the crystal structure of TiO_2_ in our experiment. The peak of TCN-2 is slightly sharpened compared to pure TiO_2_, indicating a better crystallinity than that before the combination.

**Figure 4:**
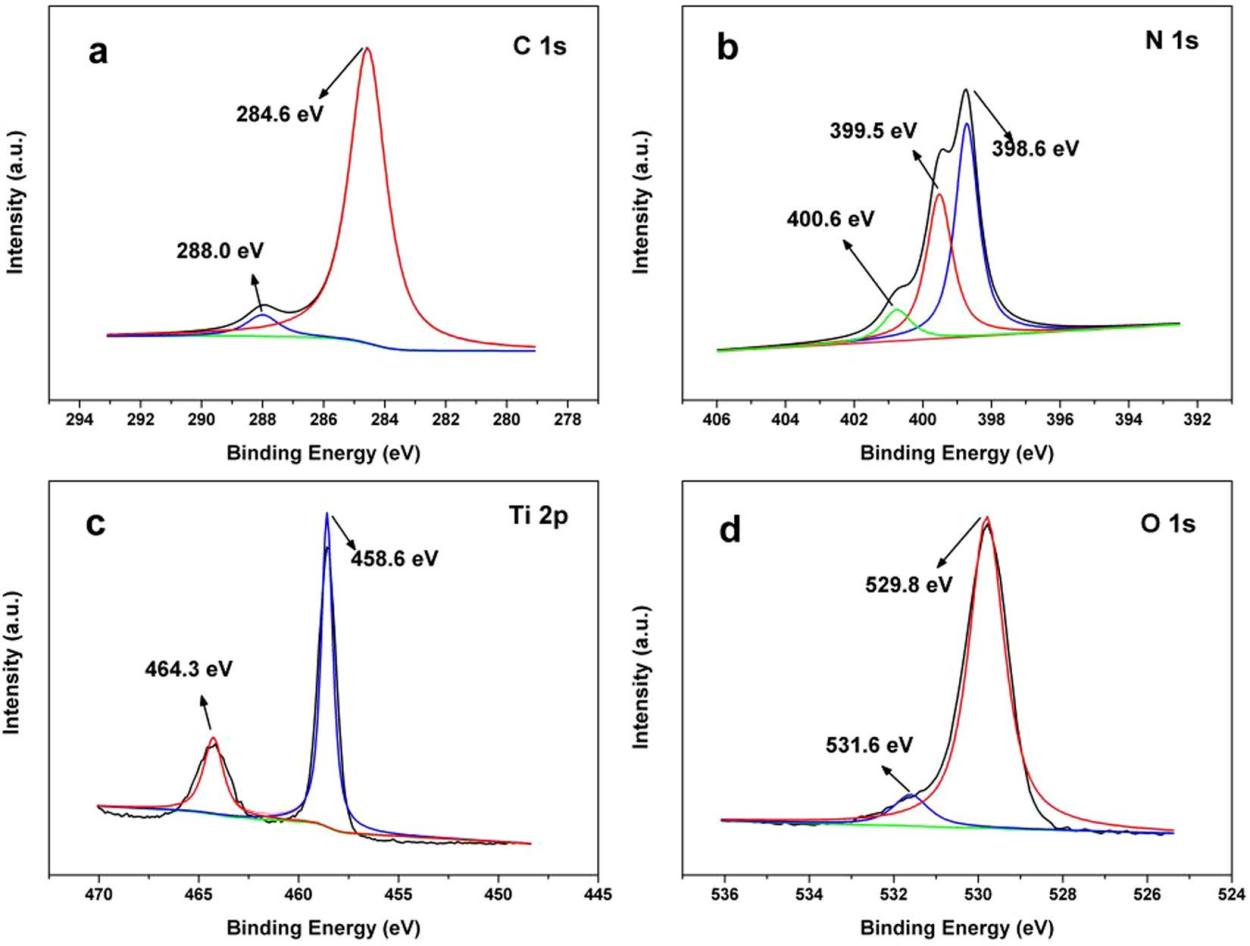
High-resolution XPS spectra of (**a**) C 1 s, (**b**) N 1 s, (**c**) Ti 2p, and (**d**) O 1s for TCN-2.

The XPS was carried out to further analyze the surface chemical composition and chemical status of elements in TCN-2 (Fig. 4). The XPS spectra of C 1 s for TCN-2, with two peaks at 284.6 and 288.0 eV are shown in Fig. 4a. The former peak is attributed to C–C, which originate from the adventitious carbon. However, the latter one is ascribed to sp^2^-hybridized carbon in N-containing aromatic ring (N–C=N)^36^. The N 1 s XPS binding energy (Fig. 4b) can be fitted into three peaks centered at 398.6, 399.5, 400.6 eV, which belong to sp^2^-hybridized nitrogen (C–N=C), the tertiary nitrogen N–(C)_3_ groups, and the free amino groups (C–N–H), respectively^37,38^. The Ti 2p spectrum has two peaks at binding energies of 458.6 (Ti 2p_3/2_) and 464.3 eV (Ti 2p_1/2_) (Fig. 4c). In Fig. 4d, the O 1 s spectrum is fitted with two peaks corresponding the Ti–O bond (529.8 eV) and O–H bond (531.6 eV), suggesting the presence of water molecule or a hydroxyl group on the surface of the TCN-2 composite microspheres^39^. The XPS results indicate that g-C_3_N_4_ was generated through melamine during the reconstruction of TiO_2_. And there are some differences compared to the g-C_3_N_4_/TiO_2_ with relatively high g-C_3_N_4_ content^40,41^, which may be due to the changes of the binding force between atoms.

#### Morphology analysis

**Figure 5:**
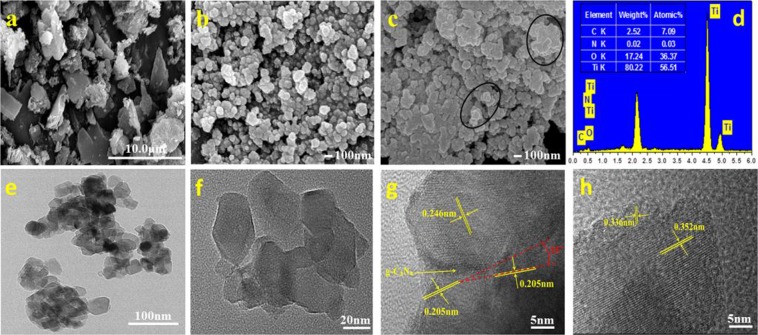
SEM images of (**a**) g-C_3_N_4_, (**b**) TiO_2_ and (**c**) TCN-2; (**d**) EDS spectra of TCN-2 sample; (**e**) TEM and (**f**–**h**) HRTEM images of TCN-2 sample.

The SEM image of the prepared g-C_3_N_4_ shows the irregular non-nanoscale structure (Fig. 5a). The photograph of spherical TiO_2_ particles is shown in Fig. 5b, and the average particle size is about 100 nm through the particle size distribution analysis. Figure 5c,d show the SEM and EDS images of TCN-2. Compared with Fig. 5b, TCN-2 is a lamellar structure (most obviously shown in the circled part of Fig. 5c), and it is more compact than pure TiO_2_ in Fig. 5b. The structural differences between TiO_2_ and TCN-2 may be the small amount of g-C_3_N_4_ that induced changes in the structure of TiO_2_. EDS spectra shows the presence of elements C, N, Ti, and O. Their weight and atomic percentages are shown in the table given as the inset of Fig. 5d. It also shows the low content of g-C_3_N_4_ in accordance with the XRD and TGA results.

**Figure 6:**
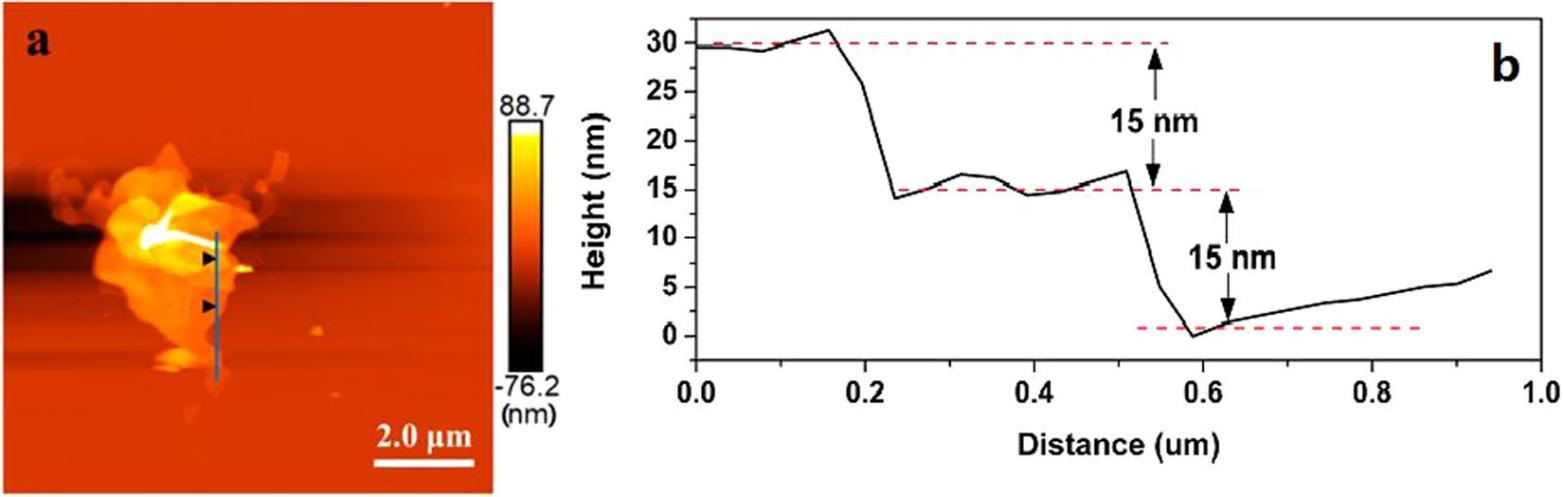
AFM images of TCN-2 hybrid with a thickness of ~15 nm.

The TEM image in Fig. 5e is in good agreement with the result of Fig. 5c, and it is further confirmed the multilayer heterointerfaces of TCN-2. In Fig. 5f,g, it is also easy to find the multilayer structure of TCN-2. In Fig. 5g, the two different lattice fringes of 0.205 nm and 0.246 nm correspond to the (200) and (004) plane of anatase TiO_2_, respectively^42^. And it could be found that the crystal surface of (200) deflected about 15°, which further confirms the interfacial misfit dislocation of TiO_2_ films between the two different planes in Fig. 5g, so that there are much more mismatch in the multilayer heterointerfaces. Additionally, some parts of the lattices of TiO_2_ are indistinct (Fig. 5g), which can be resulted from a thin layer of g-C_3_N_4_ covered with TiO_2_. Thus, it is the stratified crossed structure of TiO_2_ films induced by g-C_3_N_4_ (Figs 4g and 5f) that formed the multilayer hybrid in Fig. 5c. Figure 5h shows that the TCN-2 sample has other two lattice fringes: one is measured to be 0.352 nm matching the (101) plane of anatase TiO_2_^43^; the other one is measured to be 0.336 nm matching the (002) plane of g-C_3_N_4_^44^. The observations further suggest that g-C_3_N_4_ distributed at the boundary of TiO_2_ films, and it could be the small amount of g-C_3_N_4_ that induced the multilayer heterointerfaces of the composite. The results of AFM (Fig. 6) showed that the average interlayer distance of the multilayer heterointerfaces is about 15 nm.

From the TGA, XRD and XPS analysis, it is clear that there was a combination of g-C_3_N_4_ and TiO_2_. During the heat treatment, TiO_2_ reconstructed its crystal structure, and the g-C_3_N_4_ fabricated from melamine combined with the surface of TiO_2_ to form new interface. It is known that when the crystal surface grows with different substances, the chemical bonding direction of the surface chemisorption will be different, leading to the deflection of the crystal self-assembly growth^45–47^. During the self-assembly reconfiguration of TiO_2_, the new interface structure of g-C_3_N_4_ and TiO_2_ was formed. And the distortion of the interface leaded to the deflection of the crystal, thus reducing the interface energy to achieve the overall thermal stability, which further caused the deflection of about 15° for the crystal surface of (200). And the interfacial misfit dislocation of TiO_2_ films induced by g-C_3_N_4_ resulted in the generation of multilayer heterointerfaces. This is the major reason that caused the morphology of TCN-2 different from other structures. The above investigations strongly confirmed that the as-prepared composite including both g-C_3_N_4_ and TiO_2_ forms a heterostructure rather than a physical mixture. Therefore, the formation of a heterojunction between g-C_3_N_4_ and TiO_2_ would be a system to achieve enhanced electron-hole separation.

The structure of the TCN in our work is somewhat similar to those studied with high g-C_3_N_4_ concentrations^25,41,48,49^, which suggests that changing the amount of g-C_3_N_4_ to a small range can also form a heterogenous structure. Furthermore, the composite in our work showed some new features, such as the multilayer structure of the hybrid and the 15 nm average interlayer distance (Fig. 6). The above TGA, XRD and XPS results showed that the reconstructed TiO_2_ crystals were accompanied with melamine thermal polymerization, and the low concentration of g-C_3_N_4_ diffused to the crystal interfaces of the reconstructed TiO_2_ at 520°C, resulting in the formation of g-C_3_N_4_ with (C-N=C), (N-C=N) and N-(C)_3_ bonding characteristics.

#### Optical properties analysis

**Figure 7:**
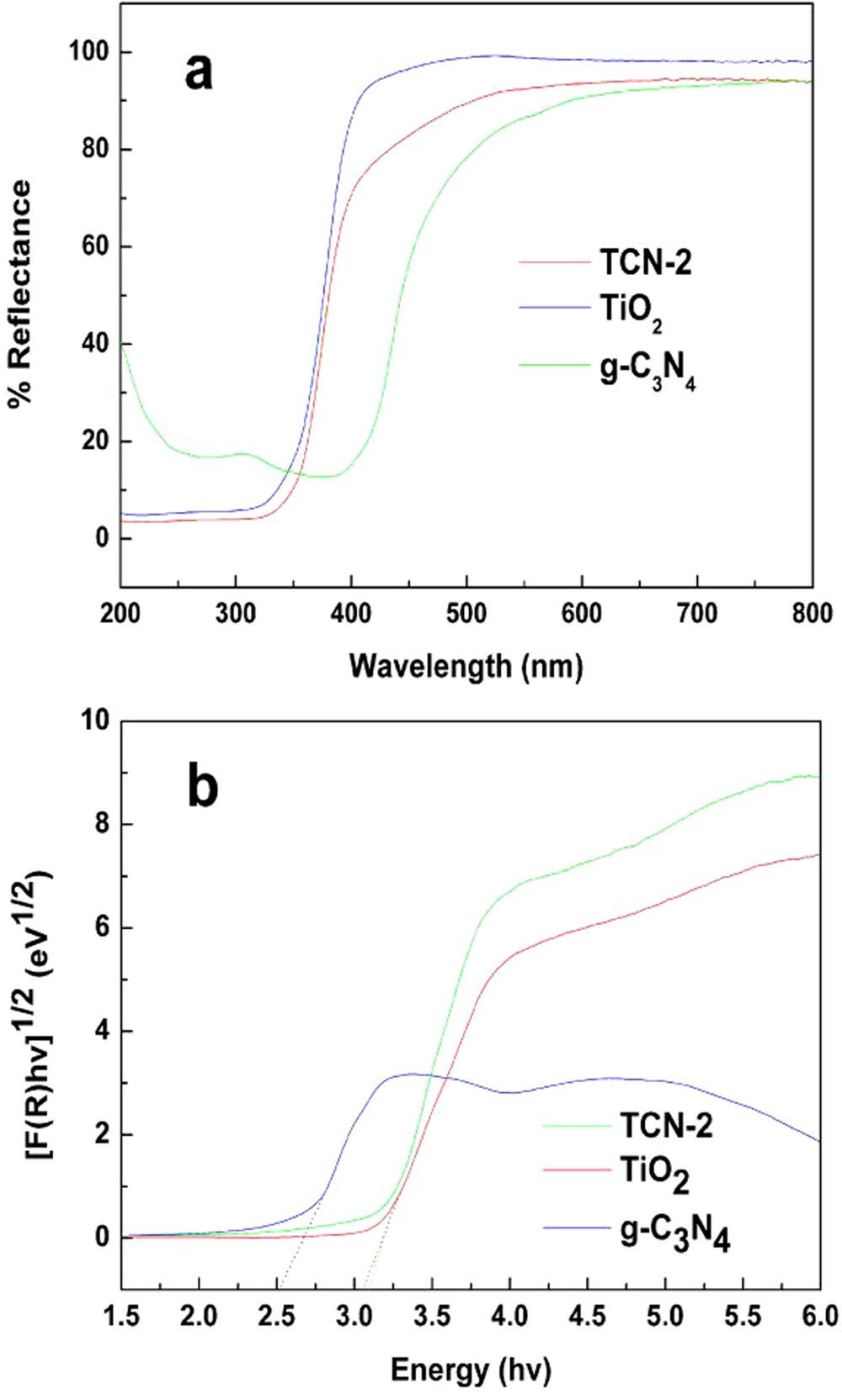
(**a**) UV–vis diffuse reflectance spectra of the photocatalysts and (**b**) their corresponding Tauc’s plots.

The UV–vis diffuse reflectance spectra of the photocatalysts are shown in Fig. 7a. And the band gap energy of the photocatalysts was calculated by the following equation^50^: ${\rm{\alpha }}\mathrm{hv}={\rm{A}}{({\rm{hv}}-{E}_{g})}^{n/2}$. In this equation, α, h, v, A, and E_g_ are absorption coefficient, Planck constant, light frequency, proportionality, and band gap energy, respectively; n keys the properties of the transition in a Semiconductor (n = 1 for direct transition, and n = 4 for indirect transition). The values of n for g-C_3_N_4_ and TiO_2_ are both 4^51,52^. The calculated results are shown in Fig. 7b, and the absorption onset of g-C_3_N_4_ was found at about 466 nm, corresponding to band gap energy of 2.66 eV. This result is consistent with other groups’ work^53^. Meanwhile, the band gap of TiO_2_ was estimated to be about 3.18 eV with the absorption edge of 389 nm. After combined with g-C_3_N_4_, the absorbance of multilayer TCN-2 is slightly extended to the visible region as the presence of g-C_3_N_4_. Combined with the analysis of Fig. 5, it could be concluded that the existence of g-C_3_N_4_ had impact on the band gap of TiO_2_.

The band edge positions of CB and VB of semiconductor could be determined by a simple approach. The valance band edge (E_VB_) and conduction band edge (E_CB_) of a semiconductor at the point of zero charge (pH_ZPC_) can be predicted by the following two equation^51^: ${E}_{VB}={\rm{\chi }}-{E}_{e}+0.5{E}_{g}$ and ${E}_{CB}={E}_{VB}-{E}_{g}$. The χ values for g-C_3_N_4_ and TiO_2_ are 4.73 eV and 5.81 eV^19^, respectively. The band gap energies of photocatalysts are shown in Table 2. Compared to the g-C_3_N_4_/TiO_2_ hybrids with high g-C_3_N_4_ content^49,54^, the band gap did not change obviously, which is due to the small amount of g-C_3_N_4_ did not influence the band gap of the composite significantly.

**Table 2:** The χ, E_CB_, E_VB_ and E_g_ for TiO_2_ and g-C_3_N_4_ at the point of zero charge.

Sample	χ (eV)	E_CB_ (eV)	E_VB_ (eV)	E_g_ (eV)
TiO_2_	5.81	−0.28	2.90	3.18
g-C_3_N_4_	4.73	−1.10	1.56	2.66

χ, E_CB_, E_VB_ and E_g_ denote absolute electronegativity, calculated CB edge, calculated VB position and band gap energy, respectively.

### Evaluation of photocatalytic activity

The degradation of RhB and MB by prepared photocatalysts was studied under UV and solar light illumination. The photocatalytic efficiencies of RhB and MB were calculated from the following expression: $\eta =[\frac{({C}_{0}-{C}_{t})}{{C}_{0}}]\times 100 \%$, where *η* is the photocatalytic efficiency; *C*_0_ is the concentration of reactant before illumination; *C*_*t*_ is the concentration of reactant after illumination time *t*. The pseudo-first-order model is expressed by the equation^50^: $\mathrm{ln}(\frac{{C}_{0}}{C})={\rm{kt}}$, where C_0_ and C are the concentrations of dye in solution at time 0 and t respectively, and k is the pseudo-first-order rate constant.

**Figure 8:**
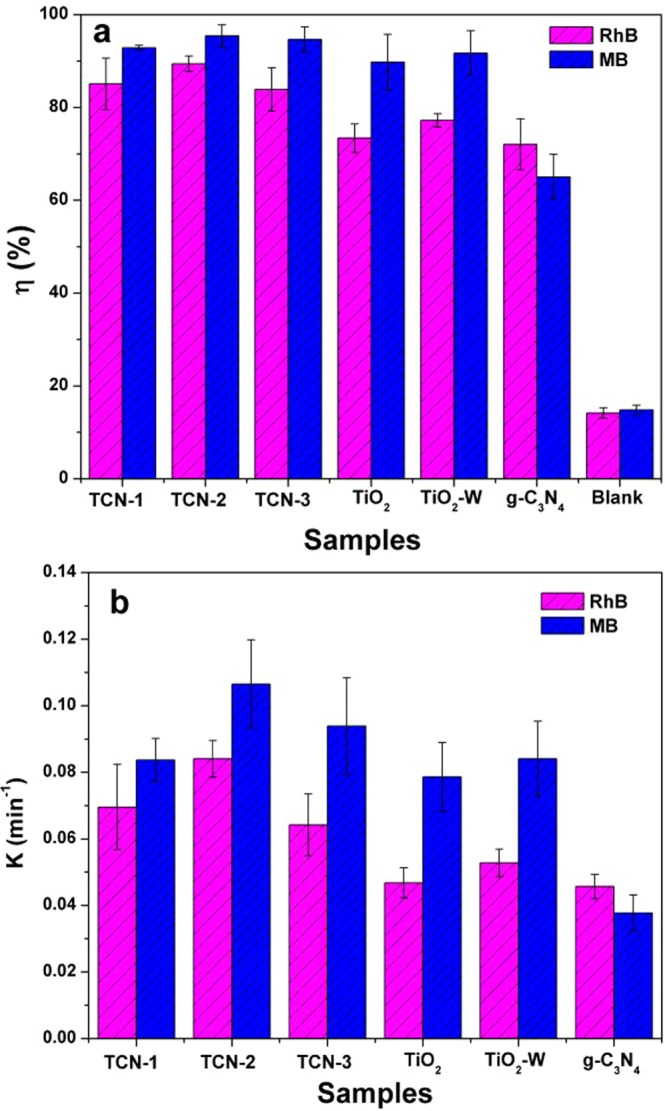
The degradation efficiency (**a**) and the kinetics (**b**) of RhB and MB by different photocatalysts under UV-light irradiation in 30 min.

**Figure 9:**
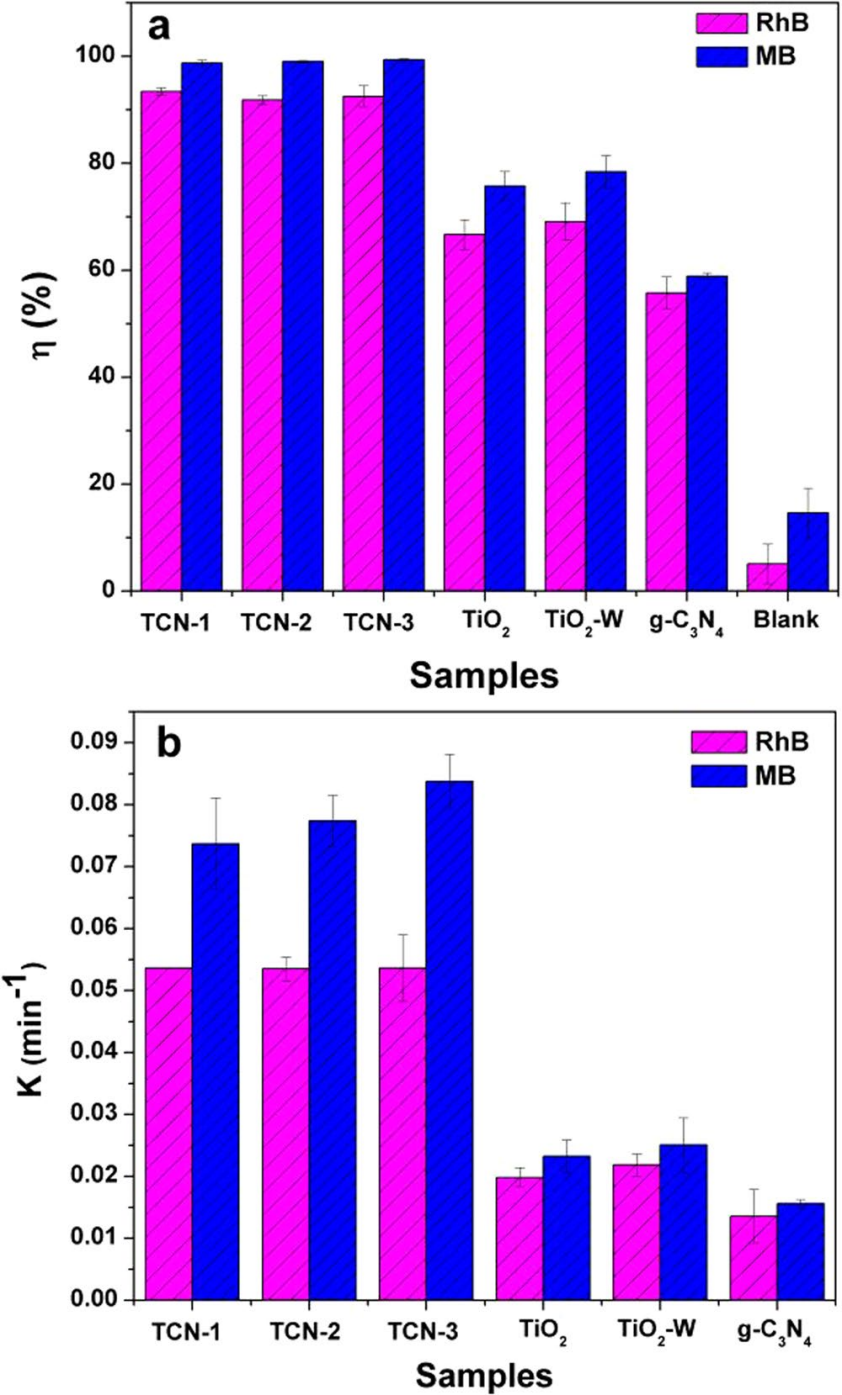
The degradation efficiency (**a**) and the kinetics (**b**) of RhB and MB by different photocatalysts under solar light irradiation in 60 min.

**Figure 10:**
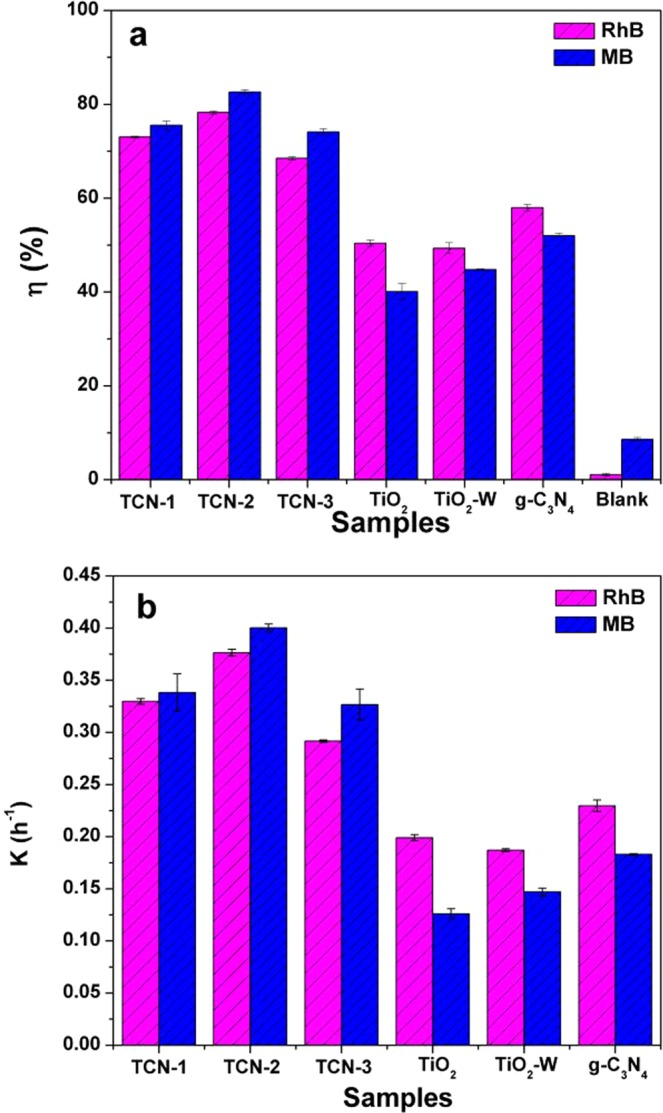
The degradation efficiency (**a**) and the kinetics (**b**) of RhB and MB by different photocatalysts under visible light (λ > 420 nm) irradiation in 4 h.

As shown in Fig. 8a, after 30 min UV illumination, the photocatalytic efficiencies of TCN-2 for RhB and MB were 89.44% and 95.48%, respectively, which were higher than that of g-C_3_N_4_ (72.09% for RhB; 65.06% for MB) and pure TiO_2_ (73.42% for RhB; 89.77% for MB). The photocatalytic efficiencies of TiO_2_–W for RhB and MB were 77.24% and 91.73%, respectively, showing a slight increase in the degradation efficiency compared to pure TiO_2_. As the photocatalytic degradation process of organic dyes follows first-order kinetics^55^, the first-order kinetics of RhB and MB degradation by the different photocatalysts are illustrated in Fig. 8b. The rate constants of TCN-2 for RhB and MB were calculated to be 0.0841 min^−1^ and 0.107 min^−1^, respectively, which were both about 2 times that of pure g-C_3_N_4_ (0.0457 min^−1^ and 0.0377 min^−1^ for RhB and MB, respectively). The rate constants of TiO_2_–W for RhB and MB were calculated to be 0.0528 min^−1^ and 0.0841 min^−1^, respectively. Moreover, the three TCN samples also showed an improved photocatalytic activity under solar light irradiation (Fig. 9). The degradation efficiencies of all TCN samples reached more than 90% in 60 min, which were much better than pure TiO_2_, TiO_2_-W and g-C_3_N_4_. And the degradation rates of TCNs for RhB and MB were more than 3 times that of pure TiO_2_, TiO_2_-W and g-C_3_N_4_. In Fig. 10, under visible light (λ > 420 nm) irradiation for 4 h, the degradation efficiencies of TCN-2 sample reached 78.27% and 82.62% for RhB and MB, respectively, which were also better than pure TiO_2_, TiO_2_-W and g-C_3_N_4_. And the degradation rates of TCN-2 for RhB and MB were more than 2 times that of pure TiO_2_, TiO_2_-W and g-C_3_N_4_. And there was no significant difference in the degradation efficiency between TiO_2_–W and TiO_2_. This further indicates that the low-content g-C_3_N_4_ doping as a surface modifying agent can improve the photocatalytic activity of TiO_2_. Although the slight change of g-C_3_N_4_ contents in the three TCN samples had a little difference in the photocatalytic performance under solar light, which may be due to the small change of hybrid’s band gap caused by the low g-C_3_N_4_ contents, the degradation efficiencies of the three samples could also further illustrate that it is feasible to improve the photocatalytic performance of TiO_2_ with low g-C_3_N_4_ content. Thus, this method would be beneficial to TiO_2_ optimization and could avoid the shortage of low yield of g-C_3_N_4_. The application of the ball-milling, microwave radiation and heating treatment method can not only effectively achieve good degradation efficiency but also reduce the cost of photocatalytic materials.

**Figure 11:**
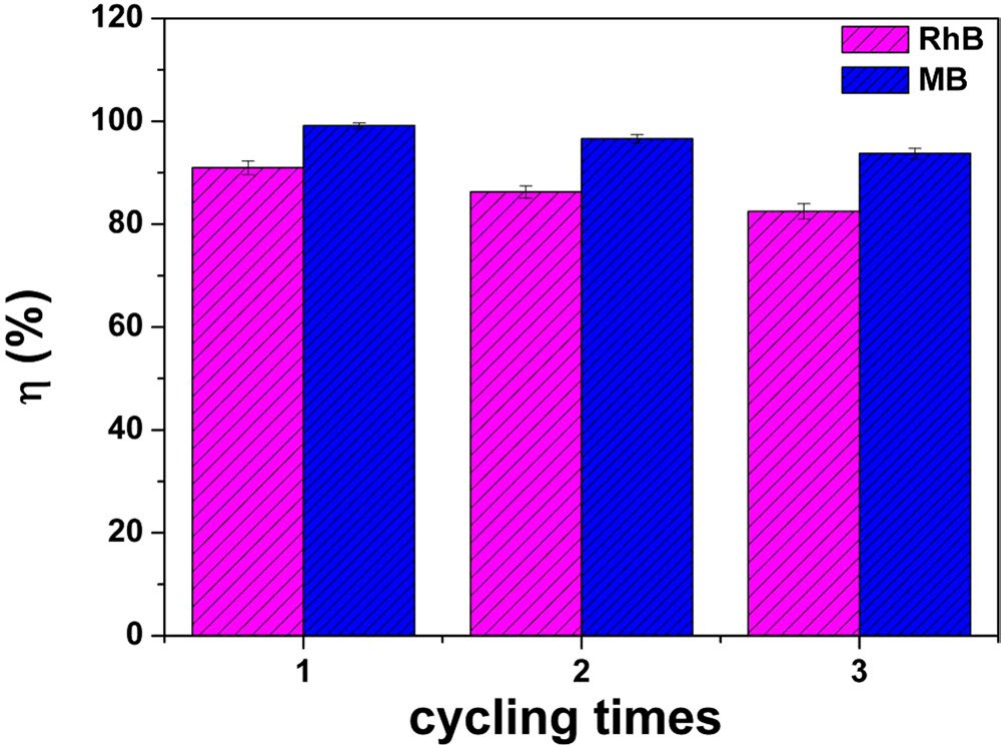
The recycling tests of TCN-2 hybrid under solar light irradiation.

Reusability is important for photocatalysts in practical applications. In the photocatalytic cycling test, the TCN-2 sample was recycled by centrifugation and then dried in an oven at 60°C for 24 h. Then, the sample was directly applied into the next recycle without any further disposal. From Fig. 11, it can be found that the photocatalytic activity of the TCN-2 hybrid was stable, and it changed little after used 3 times. Thus the compound is expected to be applied in practical photocatalysis.

### Proposed reaction mechanisms

#### PL emission spectra

**Figure 12:**
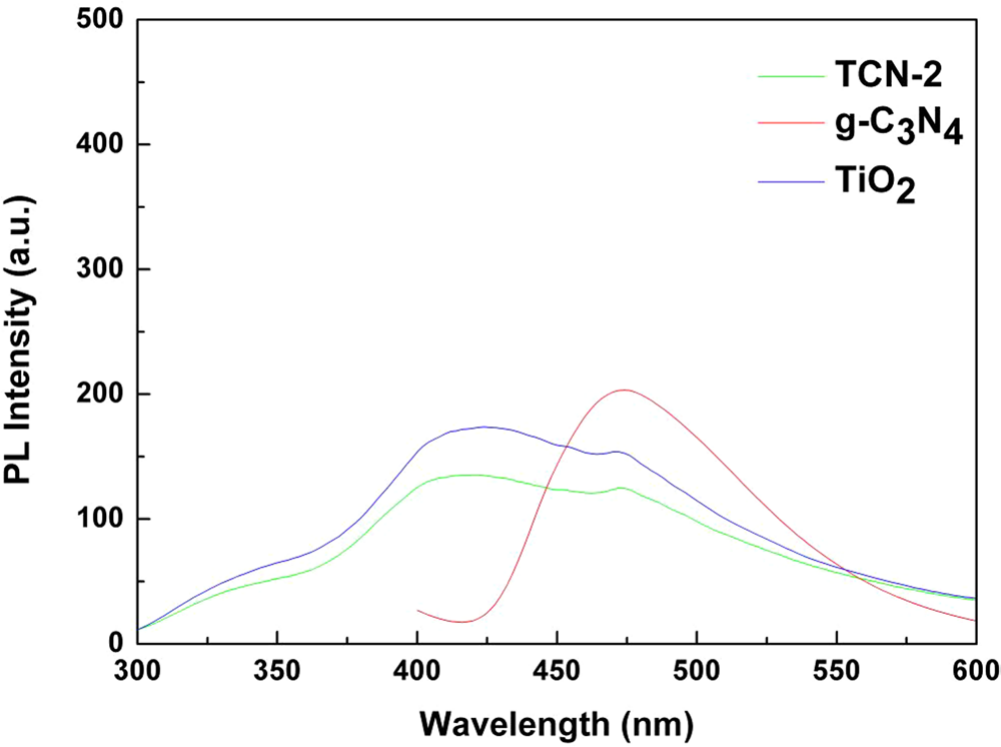
PL spectra of as-prepared g-C_3_N_4_, TiO_2_ and TCN-2 samples.

PL emission spectra can be employed to investigate the migration, transfer and separation efficiency of the photoinduced carriers in the photocatalytic reactions, due to the intensity of PL emission spectra could indicate the recombination speed of photoexcited electronhole pairs^19,51^. In Fig. 12, in the case of the pure g-C_3_N_4_ sample, the strong emission peak was at about 466 nm, which was consistent with the UV–vis DRS analysis. This strong peak is ascribed to the band-band PL phenomenon with the energy of light approximately corresponding to Huang’s work^23^. The PL spectrum of pure TiO_2_ was in the wavelength range between 350 and 550 nm, which is attributed to the emission of bandgap transition and excitonic PL that resulted from the surface oxygen vacancies and defects^56^. When compared to the pure TiO_2_ and g-C_3_N_4_, the emission spectrum of TCN-2 hybrid decreased sharply, which indicates that the e^−^–h^+^ pair recombination rate is much lower in the multilayer heterointerfaces, demonstrating the fact that g-C_3_N_4_ layers with two-dimensional π-conjugation structure could serve as an effective carrier-transfer channel and suppress the direct recombination of e^−^–h^+^ pairs in TiO_2_^22^. As the PL intensity of TCN-2 was much weaker than that of the pure TiO_2_ and g-C_3_N_4_, it is not difficult to understand that the TCN-2 sample exhibits an enhanced photocatalytic performance on the decomposition of RhB and MB.

#### Roles of reactive species

**Figure 13:**
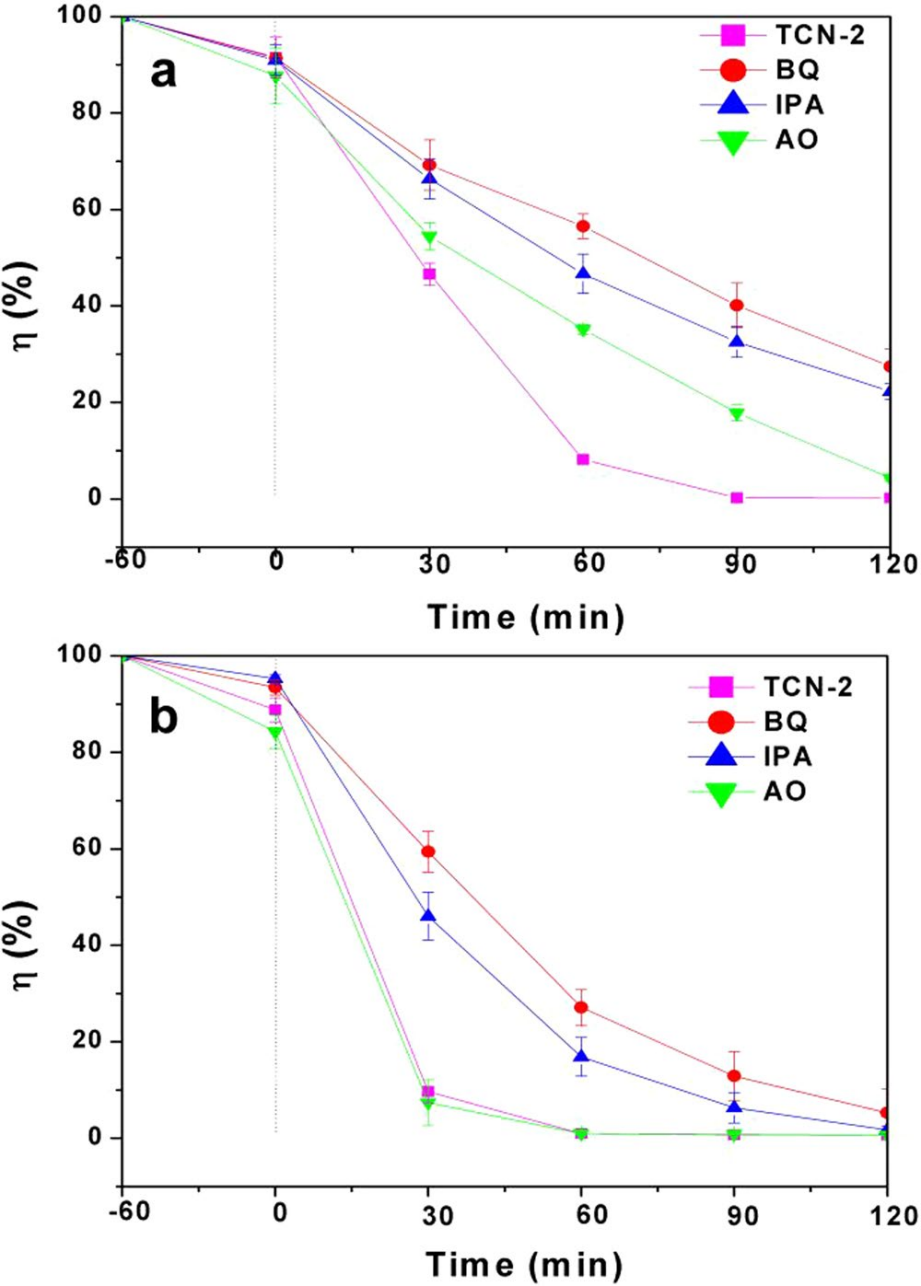
Trapping test of photogenerated holes and radicals in TCN photocatalytic system under solar light irradiation for the degradation of (**a**) RhB and (**b**) MB.

It is generally accepted that the •O_2_^−^, •OH and h^+^ are the major reactive species in the photocatalytic oxidation process^57^. In our work, the benzoquinone (BQ) was applied to reduce the superoxide radical (•O_2_^−^), the isopropanol (IPA) was employed to impair the hydroxyl radical (•OH), and the ammonium oxalate (AO) was used to remove hole (h^+^). The results are shown in Fig. 13. It could be found that, whether it is the degradation of RhB or MB, it is remarkably suppressed when the BQ and IPA was added into the reaction solutions, while the impact caused by AO was insignificant. Therefore, •O_2_^−^ and •OH are the major reactive species for TCN samples in the photocatalytic reaction system and the influencing degree is •O_2_^−^ > •OH > h^+^.

#### Photocatalytic mechanisms

**Figure 14:**
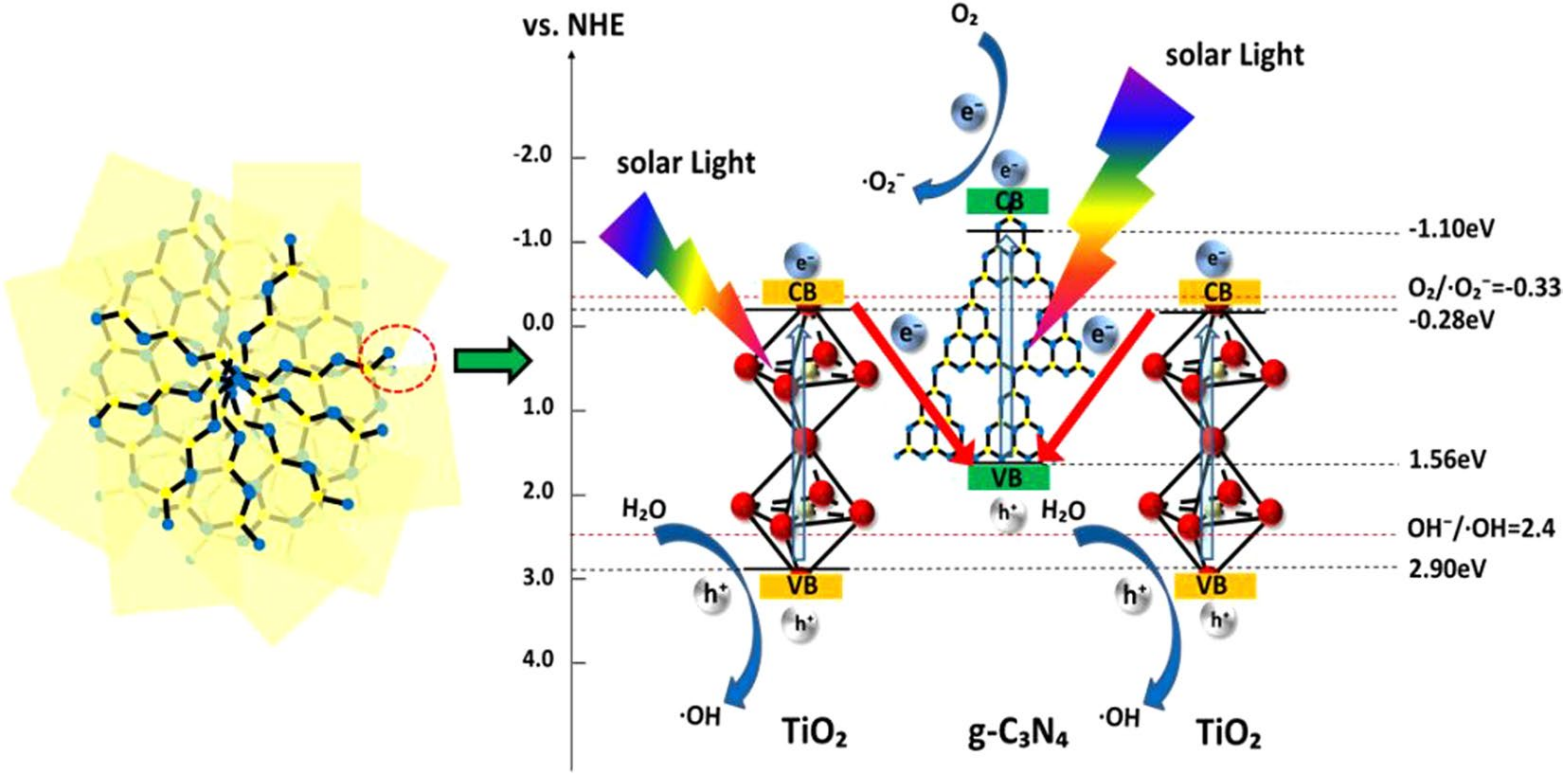
Proposed mechanism for g-C_3_N_4_/TiO_2_ photocatalysis under direct solar light irradiation.

Based on the results of the photocatalytic activities over catalysts and the trapping experiments of the photogenerated carriers, the separation processes of photoexcited electron-hole can be described in Fig. 14. From Table 2, the CB bottom of g-C_3_N_4_ (−1.10 eV) is more negative than that of TiO_2_ (−0.28 eV), nevertheless the VB top of TiO2 (2.90 eV) is more positive than that of g-C_3_N_4_ (1.56 eV). Thus, these accumulated electrons in the CB of TiO_2_ cannot reduce O_2_ to yield •O_2_^−^ (O_2_/•O_2_^−^ = −0.33 eV^8,58^), and the holes in the VB of g-C_3_N_4_ cannot oxidize OH^−^ to give •OH(OH^−^/•OH = 2.4 eV^13^). Therefore, the photocatalyst transfer scheme of charge carriers is a special Z-scheme multilayer heterointerface model rather than a traditional one^21,59,60^. Under this mechanism, the fast combination is achieved between the photoexcited holes in the VB of g-C_3_N_4_ and photoexcited electrons in the CB of TiO_2_. At the same time, the electrons in the CB of g-C_3_N_4_ with more negative potential reduce the molecular O_2_ to yield •O_2_^−^; and the holes in the VB of TiO_2_ with more positive potential oxidize OH^−^ to produce abundant •OH radicals. Furthermore, in the special crystal interfaces of the multilayer g-C_3_N_4_/TiO_2_ composite, the charge distribution has a superposition effect based on a special Z-scheme electron-hole transfer model. And from Fig. 6, several active surfaces and interfaces can be formed at the multilayer step interfaces, and the •O_2_^−^ and OH^−^ can be produced at different layers respectively, thus leading to the better photocatalytic efficiency.

## Conclusions

A novel multilayered g-C_3_N_4_/TiO_2_ composite with low g-C_3_N_4_ content was prepared via a simple ball milling, microwave radiation and heating treatment method. This method, with low temperature and simple process to produce heterojunction materials, achieved the surface modification of TiO_2_ by a small amount of g-C_3_N_4_ and feasible for industrial production. The prepared g-C_3_N_4_/TiO_2_ hybrid decorated by low g-C_3_N_4_ content has the following three features: 1) the multilayer structure shows an average interlayer distance of about 15 nm; 2) the low content of g-C_3_N_4_ fabricated from melamine spreads over the crystal surface boundary of TiO_2_ and results in the deflection of TiO_2_ films; 3) it is the deflection of different TiO_2_ crystal surfaces that further formed the multilayer heterointerfaces, leading to an improved photocatalytic activity through the degradation of RhB and MB. Furthermore, through our surface modification method, the small amount of g-C_3_N_4_ (less than 10 wt%) with the C**-**N**=**C, N**-**C=N and N-(C)_3_ bonding characteristics clustered at the crystal interfaces of the multilayer heterostructure, promoting the formation of multilayer steps with uniform thickness (15 nm). At such surface interface with special crystal interfaces and step defects, the charge distribution has a superposition effect based on a special Z-scheme electron-hole transfer model, and the •O_2_^−^ (g-C_3_N_4_) and OH^−^ (TiO_2_) can be produced at different layers respectively. Thus, the photocatalytic property of the material is effectively improved. As different preparing methods and the changes of g-C_3_N_4_ content could lead to different properties of the heterostructrues, we will continue to further explore and study in this filed.

## Supplementary information

Dataset 1

## References

[1] Chae SY (2017). Insight into Charge Separation in WO_3_/BiVO_4_ Heterojunction for Solar Water Splitting. ACS Appl. Mater. Inter..

[2] Huo R (2018). Self-assembly synthesis of BiVO_4_/Polydopamine/g-C_3_N_4_ with enhanced visible light photocatalytic performance. Mater. Res. Bull..

[3] Reshak AH (2018). Sulfide oxide XZnSO (X = Ca or Sr) as novel active photocatalytic water splitting solar-to-hydrogen energy conversion. Appl. Catal. B: Environ..

[4] Tsege EL (2017). Scalable and inexpensive strategy to fabricate CuO/ZnO nanowire heterojunction for efficient photoinduced water splitting. J. Mater. Sci..

[5] Zou, Y. *et al*. CdS quantum dots decorated ultrathin WS_2_/g-C_3_N_4_ 2D/2D heterojunction nanosheets for highly efficient photocatalytic hydrogen production under visible light. *ChemSusChem* (2018). 10.1002/cssc.201800053 29400001

[6] Bu Y (2018). Photogenerated-carrier separation along edge dislocation of WO_3_ single-crystal nanoflower photoanode. J. Mater. Chem. A.

[7] Wang R (2018). Fabrication of FTO–BiVO_4_–W–WO_3_ photoanode for improving photoelectrochemical performance: based on the Z-scheme electron transfer mechanism. J. Mater. Chem. A.

[8] Mousavi M (2016). A Novel magnetically separable g-C_3_N_4_/Fe_3_O_4_/Ag_3_VO_4_/Ag_2_CrO_4_ nanocomposites as efficient visible-light-driven photocatalysts for degradation of water pollutants. J. Mater. Sci-Mater. El..

[9] Shi L (2014). Enhanced visible-light photocatalytic activity and stability over g-C_3_N_4_/Ag_2_CO_3_ composites. J. Mate. Sci..

[10] Xiao K (2016). Mixed-calcination synthesis of Bi_2_MoO_6_/g-C_3_N_4_ heterojunction with enhanced visible-light-responsive photoreactivity for RhB degradation and photocurrent generation. Mater. Res. Bull..

[11] Tian H (2018). Sulfur- and Carbon-Codoped Carbon Nitride for Photocatalytic Hydrogen Evolution Performance Improvement. ACS Sustain. Chem. Eng..

[12] Zhou L (2016). The preparation, and applications of g-C_3_N_4_/TiO_2_ heterojunction catalysts—a review. Res. Chem. Intermed..

[13] Chen S (2015). Fabrication and characterization of novel Z-scheme photocatalyst WO_3_/g-C_3_N_4_ with high efficient visible light photocatalytic activity. Mater. Chem. Phys..

[14] Chen H (2012). Titanium dioxide photocatalysis in atmospheric chemistry. Chem. Rev..

[15] Yao T (2016). A Simple Method for the Preparation of TiO_2_/Ag-AgCl@Polypyrrole Composite and Its Enhanced Visible-Light Photocatalytic Activity. Chem. Asian J..

[16] Gu W (2017). Face-to-Face Interfacial Assembly of Ultrathin g-C_3_N_4_ and Anatase TiO_2_ Nanosheets for Enhanced Solar Photocatalytic Activity. ACS Appl. Mater. Inter..

[17] Pan C (2018). *In situ* construction of g-C_3_N_4_/TiO_2_ heterojunction films with enhanced photocatalytic activity over magnetic-driven rotating frame. Appl. Surf. Sci..

[18] Su J (2016). Self-assembly graphitic carbon nitride quantum dots anchored on TiO_2_ nanotube arrays: An efficient heterojunction for pollutants degradation under solar light. J. Hazard. Mater..

[19] Hao R (2016). Template-free preparation of macro/mesoporous g-C_3_N_4_/TiO_2_ heterojunction photocatalysts with enhanced visible light photocatalytic activity. Appl. Catal. B: Environ..

[20] Lei J (2015). Surface modification of TiO_2_ with g-C_3_N_4_ for enhanced UV and visible photocatalytic activity. J. Alloys Compd..

[21] Ma J (2016). Enhanced photocatalytic oxidation of NO over g-C_3_N_4_-TiO_2_ under UV and visible light. Appl. Catal. B: Environ..

[22] Gu L (2014). Graphitic-C_3_N_4_-hybridized TiO_2_ nanosheets with reactive {001} facets to enhance the UV- and visible-light photocatalytic activity. J. Hazard. Mater..

[23] Huang ZA (2015). Effect of contact interface between TiO_2_ and g-C_3_N_4_ on the photoreactivity of g-C_3_N_4_/TiO_2_ photocatalyst: (001) vs (101) facets of TiO_2_. Appl. Catal. B: Environ..

[24] Guo N (2018). Novel mesoporous TiO_2_@g-C_3_N_4_ hollow core@shell heterojunction with enhanced photocatalytic activity for water treatment and H_2_ production under simulated sunlight. J. Hazard. Mater..

[25] Fu M (2014). Growth of g-C_3_N_4_ Layer on Commercial TiO_2_ for Enhanced Visible Light Photocatalytic Activity. J. Nanomater..

[26] Neoh KG (2017). Surface modification strategies for combating catheter-related complications: recent advances and challenges. J. Mater. Chem. B.

[27] Méndez-Medrano MG (2016). Surface Modification of TiO_2_ with Ag Nanoparticles and CuO Nanoclusters for Application in Photocatalysis. J. Phys. Chem. C.

[28] James SL (2012). Mechanochemistry: opportunities for new and cleaner synthesis. Chem. Soc. Rev..

[29] Majid F (2014). Microwave-assisted sol–gel synthesis of BiFeO_3_ nanoparticles. J. Sol-Gel Sci. Technol..

[30] Sun Z (2018). Facile synthesis of two clay minerals supported graphitic carbon nitride composites as highly efficient visible-light-driven photocatalysts. J. Colloid Interf. Sci..

[31] Zada A (2018). Improved visible-light activities for degrading pollutants on TiO_2_/g-C_3_N_4_ nanocomposites by decorating SPR Au nanoparticles and 2,4-dichlorophenol decomposition path. J. Hazard. Mater..

[32] Zhou J (2015). Photocatalytic enhancement of hybrid C_3_N_4_/TiO_2_ prepared via ball milling method. Phys. Chem. Chem. Phys..

[33] Yu J (2013). Enhanced photocatalytic performance of direct Z-scheme g-C_3_N_4_-TiO_2_ photocatalysts for the decomposition of formaldehyde in air. Phys. Chem. Chem. Phys..

[34] Li Z (2016). Highly efficient hydrogen evolution over Co(OH)_2_ nanoparticles modified g-C_3_N_4_ co-sensitized by Eosin Y and Rose Bengal under Visible Light Irradiation. Appl. Catal. B: Environ..

[35] Liu Y (2015). Enhanced visible-light photocatalytic activity of Z-scheme graphitic carbon nitride/oxygen vacancy-rich zinc oxide hybrid photocatalysts. Chinese J. Catal..

[36] Li X (2015). Hydrogenated Defects in Graphitic Carbon Nitride Nanosheets for Improved Photocatalytic Hydrogen Evolution. J. Phys. Chem. C.

[37] Kuang P-Y (2015). g-C_3_N_4_ decorated ZnO nanorod arrays for enhanced photoelectrocatalytic performance. Appl. Surf. Sci..

[38] Chen Y (2014). Construction of heterostructured g-C_3_N_4_/Ag/TiO_2_ microspheres with enhanced photocatalysis performance under visible-light irradiation. ACS Appl. Mater. Inter..

[39] Kim J (2016). Individually carbon-coated and electrostatic-force-derived graphene-oxide-wrapped lithium titanium oxide nanofibers as anode material for lithium-ion batteries. Electrochim. Acta.

[40] Zhou D (2016). Facile Construction of g-C_3_N_4_ Nanosheets/TiO_2_ Nanotube Arrays as Z-Scheme Photocatalyst with Enhanced Visible-Light Performance. ChemCatChem.

[41] Zang Y (2014). Hybridization of brookite TiO_2_ with g-C_3_N_4_: a visible-light-driven photocatalyst for As^3+^ oxidation, MO degradation and water splitting for hydrogen evolution. J. Mater. Chem. A.

[42] Ye L (2013). Synthesis of anatase TiO_2_ nanocrystals with {101}, {001} or {010} single facets of 90% level exposure and liquid-phase photocatalytic reduction and oxidation activity orders. J. Mater. Chem. A.

[43] Gun Y (2015). Joint Effects of Photoactive TiO_2_ and Fluoride-Doping on SnO_2_ Inverse Opal Nanoarchitecture for Solar Water Splitting. ACS Appl. Mater. Inter..

[44] Li G (2015). Enhanced visible-light-driven photocatalytic inactivation of Escherichia coli using g-C_3_N_4_/TiO_2_ hybrid photocatalyst synthesized using a hydrothermal-calcination approach. Water Res..

[45] Chun Y (2004). Ab initio total energy study of ZnO adsorption on a sapphire (0001) surface. Phys. Rev. B.

[46] Huang P (2015). A DFT study of STO adsorption on GaN (0001) surface. Chem. Phys. Lett..

[47] Yang C (2010). Modelling and simulation of reaction mechanisms in early growth of STO thin films from ab initio calculations. Comp. Mater. Sci..

[48] Fu M (2013). A Cost-Effective Solid-State Approach to Synthesize g-C_3_N_4_ Coated TiO_2_ Nanocomposites with Enhanced Visible Light Photocatalytic Activity. Int. J. Photoenergy.

[49] Tong Z (2015). Biomimetic fabrication of g-C_3_N_4_/TiO_2_ nanosheets with enhanced photocatalytic activity toward organic pollutant degradation. Chem. Eng. J..

[50] Zhang J (2014). Design of a direct Z-scheme photocatalyst: preparation and characterization of Bi_2_O_3_/g-C_3_N_4_ with high visible light activity. J. Hazard. Mater..

[51] Chen S (2014). Study on the separation mechanisms of photogenerated electrons and holes for composite photocatalysts g-C_3_N_4_-WO_3_. Appl. Catal. B: Environ..

[52] Sridharan K (2013). Novel visible light active graphitic C_3_N_4_–TiO_2_ composite photocatalyst: Synergistic synthesis, growth and photocatalytic treatment of hazardous pollutants. Appl. Catal. B: Environ..

[53] Giannakopoulou T (2017). Tailoring the energy band gap and edges’ potentials of g-C_3_N_4_/TiO_2_ composite photocatalysts for NOx removal. Chem. Eng. J..

[54] Zhao S (2012). g-C_3_N_4_/TiO_2_ hybrid photocatalyst with wide absorption wavelength range and effective photogenerated charge separation. Sep. Purif. Technol..

[55] Bansal P (2015). Comparative study of catalytic activity of ZrO_2_ nanoparticles for sonocatalytic and photocatalytic degradation of cationic and anionic dyes. Chem. Eng. J..

[56] Yu J (2011). Enhanced photocatalytic activity of mesoporous TiO_2_ aggregates by embedding carbon nanotubes as electron-transfer channel. Phys. Chem. Chem. Phys..

[57] Li W (2012). Evidence for the Active Species Involved in the Photodegradation Process of Methyl Orange on TiO_2_. J. Phys. Chem. C.

[58] Gao S (2017). Photocatalytic removal of tetrabromobisphenol A by magnetically separable flower-like BiOBr/BiOI/Fe_3_O_4_ hybrid nanocomposites under visible-light irradiation. J. Hazard. Mater..

[59] Wei Z (2017). Photoelectrocatalytic degradation of phenol-containing wastewater by TiO_2_/g-C_3_N_4_ hybrid heterostructure thin film. Appl. Catal. B: Environ..

[60] Wei X (2016). Facile *in situ* synthesis of plasmonic nanoparticles-decorated g-C_3_N_4_/TiO_2_ heterojunction nanofibers and comparison study of their photosynergistic effects for efficient photocatalytic H2 evolution. Nanoscale.

[61] Wang J (2014). A Synthesis of g-C_3_N_4_/TiO_2_ with enhanced photocatalytic activity for H_2_ evolution by a simple method. Int. J. Hydrogen Energ..

[62] Yan H (2011). TiO_2_–g-C_3_N_4_ composite materials for photocatalytic H_2_ evolution under visible light irradiation. J. Alloys Compd..

