# Author Correction: Novel multilayer TiO_2_ heterojunction decorated by low g-C_3_N_4_ content and its enhanced photocatalytic activity under UV, visible and solar light irradiation

**DOI:** 10.1038/s41598-019-48366-z

**Published:** 2019-09-27

**Authors:** Yizheng Wang, Jiang Yu, Weidong Peng, Jing Tian, Chun Yang

**Affiliations:** 1 College of Architecture and Environment, Sichuan University, Chengdu, 610065 China; 2 Institute of New Energy and Low Carbon Technology, Sichuan University, Chengdu, 610065 China; 3 College of Chemistry and Materials Science, Sichuan Normal University, Chengdu, 610068 China; 4 Computational Visualization and Virtual Reality Key Laboratory of Sichuan Province, Chengdu, 610068 China

Correction to: *Scientific Reports* 10.1038/s41598-019-42438-w, published online 11 April 2019

In the original version of this Article, Chuan Yang was incorrectly listed as the corresponding author. The correct corresponding author for this Article is Jiang Yu. Correspondence and request for materials should be addressed to yujianggz@163.com. This error has now been corrected in the HTML and PDF versions of the Article.

